# Dysregulated Tear Film Proteins in Macular Edema Due to the Neovascular Age-Related Macular Degeneration Are Involved in the Regulation of Protein Clearance, Inflammation, and Neovascularization

**DOI:** 10.3390/jcm10143060

**Published:** 2021-07-10

**Authors:** Mateusz Winiarczyk, Dagmara Winiarczyk, Katarzyna Michalak, Kai Kaarniranta, Łukasz Adaszek, Stanisław Winiarczyk, Jerzy Mackiewicz

**Affiliations:** 1 Department of Vitreoretinal Surgery, Medical University of Lublin, 20-079 Lublin, Poland; jerzymackiewicz@umlub.pl; 2 Department of Epizootiology, University of Life Sciences of Lublin, 20-400 Lublin, Poland; winiarczykdm@gmail.com (D.W.); artica@wp.pl (K.M.); lukasz.adaszek@up.lublin.pl (Ł.A.); genp53@interia.pl (S.W.); 3 Department of Ophthalmology, University of Eastern Finland and Kuopio University Hospital, 70211 Kuopio, Finland; kai.kaarniranta@uef.fi

**Keywords:** age-related macular degeneration, AMD, proteomics, tear film, tear film proteome, protein clearance, neovascularization, neovascular AMD

## Abstract

Macular edema and its further complications due to the leakage from the choroidal neovascularization in course of the age-related macular degeneration (AMD) is a leading cause of blindness among elderly individuals in developed countries. Changes in tear film proteomic composition have been reported to occur in various ophthalmic and systemic diseases. There is an evidence that the acute form of neovascular AMD may be reflected in the tear film composition. Tear film was collected with Schirmer strips from patients with neovascular AMD and sex- and age-matched control patients. Two-dimensional electrophoresis was performed followed by MALDI-TOF mass spectrometry for identification of differentially expressed proteins. Quantitative analysis of the differential electrophoretic spots was performed with Delta2D software. Altogether, 11 significantly differentially expressed proteins were identified; of those, 8 were downregulated, and 3 were upregulated in the tear film of neovascular AMD patients. The differentially expressed proteins identified in tear film were involved in signaling pathways associated with impaired protein clearance, persistent inflammation, and neovascularization. Tear film protein analysis is a novel way to screen AMD-related biomarkers.

## 1. Introduction

Age-related macular degeneration (AMD) is a leading cause of blindness in elderly patients in developed countries. The incidence of AMD is expected to increase by over 50% in the next 20 years [1]. AMD affects central vision by evoking metamorphopsia, reading problems, and eventually legal blindness in its end stage. AMD can be divided into wet (neovascular) and dry (atrophic) forms. Usually, atrophic AMD progresses slowly over years, while neovascular AMD with the presence of subretinal fluid and macular edema can develop in weeks due to the progressive growth of pathological choroidal vessels. Currently, there is no established treatment protocol for atrophic AMD, but anti-vascular endothelial growth factor (VEGF) intravitreal injections are a treatment of choice for neovascular AMD. Although our awareness of AMD etiopathology has significantly improved in the past decade, the exact mechanisms underlying the disease are still vague. The cellular mechanisms of AMD are known to be linked to chronic oxidative stress (OS), autophagy impairment, and inflammation that can ultimately lead to retinal pigment epithelium (RPE) cell and photoreceptor death [2,3]. AMD development is also strongly associated with genetic variations and mutations in the complement system, as well as with many environmental risk factors, such as smoking, hypercholesterolemia, arteriosclerosis, obesity, and unhealthy diet consumption [4].

Tear film is a mixture of lipids, water, and mucin that covers the surface of the eye. It protects against an environment-evoked irritation and smooths the corneal surface to improve the refractive effect. Tear film is produced by lacrimal and accessory glands, as well as by meibomian glands and goblet cells [5]. Since tear film is readily accessible, it has been analyzed in many clinical studies on dry eye syndrome, diabetes, Parkinson’s disease, multiple sclerosis, and cancer [6,7,8,9,10,11,12].

Previous studies concerning proteomic changes that occur over the course of AMD have focused mainly on the aqueous humor, the vitreous body, donor retinas, and blood [13,14,15,16,17,18,19,20,21,22,23,24,25,26,27,28,29]. In each of these studies, significant differences in the expression of certain proteins have been discovered. The identified proteins are usually involved in metabolic pathways associated with AMD. We reviewed most of the recent developments in AMD proteomic research in our previous manuscript, in which we sought to determine whether the pathological process in the macula can result in tear film proteome changes [30]. Although we discovered various differentially expressed proteins, we were not able to perform quantitative analysis. In this study, we analyzed tear film samples from neovascular AMD patients to identify and quantify proteins that were differentially expressed between a neovascular AMD group and a control group.

## 2. Materials and Methods

The study was approved by the Bioethical Committee of the Medical University of Lublin under declaration number KE-0254/238/2015. Informed consent was obtained from every individual enrolled in the study. The purpose and design of the study, as well as its possible complications, were explained to every patient, and written consent was obtained. All experiments followed the provisions of the Declaration of Helsinki.

In total, 30 patients were included in the study: 15 patients in the neovascular AMD group, and 15 in the control group. The sex distribution was similar between the groups. All criteria-satisfying patients underwent a full ophthalmic examination by the same ophthalmologist (MW) that consisted of a visual acuity test, slit lamp examination, intraocular pressure (IOP) measurement, spectral domain optical coherent tomography (SDOCT, Copernicus, Optopol Technologies, Zawiercie, Poland), and fluorescein angiography or optical coherence tomography angiography (angio-OCT, RTVue XR 100 Avanti, Optovue, Fremont, CA, USA). The tear film break-up times (BUTs) were within normal limits (over 10 s), and all the patients had Schirmer test results of greater than 15 mm in 5 min.

The inclusion criteria for the AMD group were as follows: active form of disease featuring choroidal neovascularization (CNV) on fluorescein angiography or angio-OCT in at least one eye and the presence of subretinal fluid.

The exclusion criteria were any ocular surface diseases that would disturb the results, e.g., dry eye syndrome, eye surface disorders, diabetic retinopathy, glaucoma, and previous ocular surgery except for cataract extraction. Additionally, any moderately advanced or advanced stage of any systemic disease, such as poorly controlled hypertension, cardiovascular disease, or autoimmune disorder, was an exclusion criterion.

The neovascular AMD group consisted of 15 patients, comprising 7 men and 8 women, with a mean age of 76.4 years (SD = 5.6). On slit lamp examination, the patients presented with an active form of AMD in at least one eye with subretinal fluid presence. All of them had previously been treated with anti-VEGF therapy in one or both eyes. Environmental risk factors, smoking, and systemic diseases were assessed. The control subjects were recruited from among patients who qualified for standard cataract surgery. The control group consisted of 15 patients, comprising 8 men and 7 women, with a mean age of 76.1 years (SD = 3.9). The mean IOP was within normal limits (8–21 mm Hg) in all patients. 

For a statistical analysis of sex difference, a chi-square test on the contingency table with control and AMD on one side and male and female on the other side, making a 2 × 2 table, and assuming the null hypothesis about independence of health from gender. Comparing the chi-square-calculated parameter with the tabular value, we dealt with independent variables, meaning that the gender composition of the groups was neutral. According to the age comparison between the groups, we performed a *t*-test to determine the difference in the means. In this case (group one mean: 76.4 ± 5.4; group two: 76.1 ± 3.9), the *p*-value equals 0.8782. By conventional criteria, this difference is not considered to be statistically significant. To confirm that the groups were statistically similar, we preformed the Shapiro–Wilk test of normality. We accepted that H0 assumed that the data in the control and AMD groups were normally distributed. We also accepted H0 in the *t*-test, which meant that the average of 1′s population was considered to be equal to the average of the 2′s population. In other words, the difference between the average of the 1 and 2 populations was not big enough to be statistically significant.

Detailed information about the study and the control groups are presented in Supplementary Materials Tables S1 and S2.

### 2.1. Sample Preparation

Tear film was collected from each eye onto a Schirmer strip (TearFlo, HUB Pharmaceuticals LLC, Scottsdale, AZ, USA) [1,2]. Each collection was performed by the author M.W. in the morning hours between 8 and 11 a.m. If fluorescein angiography was performed, the material was always collected beforehand. Sterile gloves were always used by the investigator. The Schirmer strips were placed into the lower conjunctival sacs of both eyes at the one-third point of the eyelid as measured from the nasal canthus without anesthesia. There is currently no consensus about which method of collection should be used for proteomic analysis [3,4,5,6]. After the strips were held in place for 5 min, they were removed, transferred to 1.5-mL Eppendorf tubes without buffer, and immediately frozen at −80 °C. Next, the proteins were extracted in urea buffer for 3 h. Extraction was carried out at 4 °C in the presence of protease inhibitor cocktail (P8340, Sigma Aldrich, St. Louis, MO, USA). The cocktail contained 104 mM 4-(2-aminoethyl)benzenesulfonyl fluoride hydrochloride (AEBSF), 80 μM aprotinin, 4 mM bestatin, 1.4 mM E-64, 2 mM leupeptin, and 1.5 mM pepstatin A. Each of these components has specific inhibitory properties. AEBSF and aprotinin inhibit serine proteases, including trypsin, chymotrypsin, and plasmin, among others; bestatin inhibits aminopeptidases; E-64 inhibits cysteine proteases; leupeptin inhibits both serine and cysteine proteases; and pepstatin A inhibits acid proteases (according to the Sigma–Aldrich specification sheet). After extraction, the strips were removed, and the extracts were centrifuged at 1844× *g* for 10 min at 4 °C. The obtained supernatants were collected and stored at −80 °C.

### 2.2. Protein Purification and Precipitation

The concentrations of the proteins were measured by a spectrophotometric method (MaestroNano Micro-Volume Spectrophotometer). Samples containing 150 µg of proteins were transferred into 1.5-mL microcentrifuge tubes and diluted with water to a final volume of 100 µL. Using a ReadyPrep 2-D Cleanup Kit (Bio-Rad, Hercules, CA, USA) the protein pellets were obtained and resuspended by adding 300 µL of rehydration sample buffer (Bio-Rad). The supernatants were applied directly to immobilized pH gradient (IPG) strips (17 cm, pH 3¨C10, linear pH gradient, Bio-Rad).

After 12 h of gel rehydration the isoelectric focusing was performed at 60 kVh with a current limit of 50 µA per strip (Hoefer IEF100). Before second-dimension separation, the IPG strips were equilibrated in two equilibration buffers (50 mM Tris-HCl, pH 8.8, 6 M urea, 30% glycerol, 2% sodium dodecyl sulfate (SDS)). The first buffer contained dithiothreitol (2%), while the second buffer contained iodoacetamide (2.5%) instead of dithiothreitol. The duration of each equilibration step was 15 min. The second dimension of electrophoretic separation was conducted using 12.5% polyacrylamide gels in a Bio-Rad PROTEAN II xi Cell (Bio-Rad). Vertical separation was performed at 600 V/50 mA/30 W in 0.025 M Tris/Gly buffer (pH 8.3). After electrophoretic separation, the proteins were silver stained in accordance with the methods of Shevchenko et al. [7].

### 2.3. Preparation of Proteins for MALDI Identification

The spots of interest were excised from the gels by scalpel, transferred into microtubes, washed with H_2_O, and distained. After that, dithiothreitol reduction and iodoacetamide alkylation were performed. The gel pieces were covered with trypsin solution containing 50 mM ammonium bicarbonate and placed in an autoclave overnight to digest at 37 °C. Next, the peptides were extracted from the gel pieces with 50 μL of acetonitrile (ACN):H_2_O:trifluoroacetic acid (TFA) (50:45:5) solution. Extraction was performed in an ultrasonic bath at room temperature and was repeated three times (each step lasted 15 min). The extracts were collected and concentrated in a CentriVap (Labconco, Kansas City, MO, USA). The obtained peptide pellets were dissolved in 10 µL of 0.1% TFA and purified with ZipTip Sample Prep Pipette Tips (0.2 μL of C18 iod, Merck, Darmstadt, Germany) in accordance with a standard procedure.

### 2.4. MALDI Analysis

Finally, 1 μL of each purified peptide sample was spotted onto an AnchorChip MALDI plate with hydrophobic coating and calibrant anchors. Next, 1 μL of alpha-cyano-4-hydroxycinnamic acid (HCCA, Bruker, Billerica, MA, USA) matrix solution was pipetted onto the dry peptide sample. A peptide calibration standard (Peptide Calibration Standard II, Bruker) was spotted on the calibrant spots. The mass spectra were recorded in active positive reflection mode by an Ultraflex III MALDI-TOF/TOF spectrometer (Bruker). All spectra were collected within the 700–4000 m/z range. The collected spectra were smoothed (Savitsky–Golay method) and the baseline corrected (Top Hat baseline algorithm) in flexAnalysis 3.0 software (Bruker). A list of peaks in the range of 700–4000 m/z for a signal-to-noise ratio greater than 3 was also generated in flexAnalysis 3.0. After removal of impurities, the final peak list was transferred to BioTools 3.2 (Bruker) and compared with Mascot 2.2 software using the Swiss–Prot database. Other parameters were set as follows: the maximum error in both MS and MS/MS was 0.3 Da; the obligatory modification was carbamidomethylation of cysteine; and the possible modifications were methionine oxidation, serine, and threonine phosphorylation, methionine dioxidation, and protein *N*-terminal acetylation. Results with scores above 56 were considered statistically significant. The peptide mass fingerprint spectra were analyzed in MS/MS mode to confirm the amino acid sequences.

### 2.5. Visual and Statistical Analysis

The stained gels were scanned using a GE Image Scanner III (GE Healthcare, Warsaw, Poland) and further processed by Delta2D software (version 4.7, DECODON). The Delta2D software enabled quantitation of the spots and creation of protein expression profiles. The utilized program uses gel image warping (correction of positional spot variations and matching of images) to create a so-called fused image. This image is a proteome map containing every protein spot obtained on every gel during the whole experiment. After the fused image was created, the spots were detected. False-negative and false-positive protein spots were determined manually. To calculate the expression ratios (Rts; spot volumes relative to the group means), a quantitation table was generated, and the volume-normalized values were statistically analyzed. In this experiment, the mean volume of a given spot in the control group was the denominator of the Rt parameter.

In the case of gel statistical analysis after normalization, we used a *t*-test for two analyzed groups with *p*-values based on t-distribution and alpha (overall all threshold *p*-value): 0.05. We took a Rt value greater than 1.5 as overexpressed and below 0.67 as suppressed.

Differences in protein expression between the test groups were analyzed by a *t*-test with statistical software built into Delta2D; a *p*-value ≤ 0.05 was considered to indicate significance. The *p*-value of 0.05 was two-sided (α/2 = 0.025 both sides). Only spots with significant differential expression between the neovascular AMD group and the control group, and with spot Rts higher than 1.5 (upregulated) or lower than 0.67 (downregulated), were selected for protein identification.

## 3. Results

Altogether, samples from 15 patients with neovascular AMD and 15 control patients were included in the proteomic analysis. Differences in protein expression levels between the two groups were identified using two-dimensional gel electrophoresis (2DE) followed by MALDI-TOF MS.

We chose groups to be as similar to each other as possible in terms of age, disease, and gender. In the AMD group, the mean age was 76.4 years ± 5.4. Patients who took part in the study were mostly smokers (73%) with systemic diseases (40% had one disease, 33% had two). This group of 15 patients consisted of 7 men (47%) and 8 females (53%). The control group was similar: The mean age was 76.1 ± 3.8, and 73% were also smokers. When it comes to the occurrence of systematic diseases, the numbers are also analogous: 40% had one disease, 33% had two. There was a minimal sex difference: the control group of 15 individuals consisted of 8 men (53%) and 7 females (47%).

**Figure 1:**
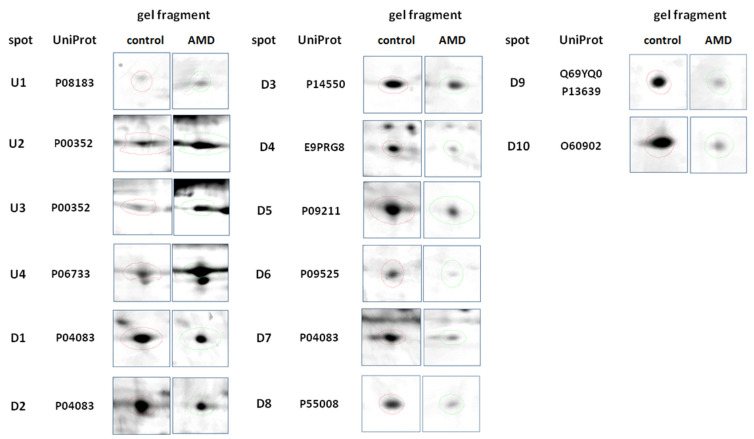
Representative 2DE gel spots of significantly (*p* ≤ 0.05) differentially expressed proteins in Table 2. D software (version 4.7, DECODON, Greifswald, Germany). Left column represents the control group, and the right column represents the AMD group.

**Figure 2:**
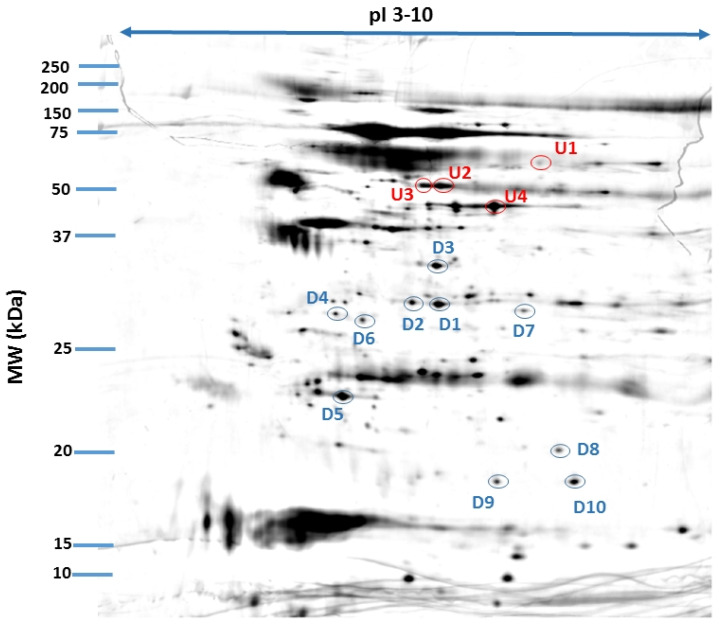
Fused image showing the condensed spot patterns from the experiment. The differentially expressed proteins in the neovascular AMD group versus the control group are marked. Upregulated proteins are indicated in red, and downregulated proteins are indicated in blue. The proteins were separated in the first dimension by isoelectric focusing over an isoelectric point (pI) range of 3–10. The second dimension was performed using 12.5% SDS polyacrylamide gels. The gels were silver stained, digitized, and processed in Delta2D software (version 4.7, DECODON).

We found 469 proteins in the analyzed tear film samples. Among those, we focused only on the differential electrophoretic spots. Bioanalytical software revealed that 31 spots exhibited significant differences between groups, and 14 spots fulfilled the Rt criteria of greater than 1.5 (upregulated) or less than 0.67 (downregulated), in three consecutive repetitions. Fourteen of the spots were positively identified. From those, 11 proteins were eventually identified, as Annexin A1 was recognized 3 times, and Retinal dehydrogenase twice. The same proteins occurring in different points of gel is a common finding. Spot multiplicity is mostly a result of post treatment modifications, which give a particular shift in pI and molecular weight. In addition, despite using cocktail protease inhibitors and DTT, protein cleavage or aggregation can happen. Table 1 contains a list of the protein names, encoding genes, UniProt base accession numbers, and Rt values. With regard to the Rts for the group means of relative spot volumes, the volume of a given spot in the control group was used as the denominator of the Rt parameter (Rt > 1.5, overexpression; Rt < 0.67, suppression). According to the results obtained with the Delta2D program, 8 of the 11 proteins were assigned to downregulated, and 3 of the 11 proteins were upregulated (Figure 1; Table 2). Figure 2 shows a fused image of 2DE gels with differentially expressed proteins in the AMD group versus the control group. 

## 4. Discussion

Currently, AMD is viewed as a disease involving impairment of multiple cellular processes; its exact pathogenesis remains unclear. Here, we found that proteins isolated from the tears of neovascular AMD patients were associated with oxidative stress, proteostasis regulation, inflammation, and neovascularization.

In our previous study [8], we identified 342 proteins that were differentially expressed in both types of AMD—atrophic and neovascular. We were, at that point in time, unable to perform a quantitative analysis of the obtained data. In the current manuscript, we quantified the identified proteins. This made it possible to pinpoint the proteins that could be more relevant for the disease progress. We also obtained a larger and more homogenous group—all of our patients presented an active stage of neovascular AMD.

### 4.1. Oxidative Stress

Oxidative stress (OS) occurs when there is an imbalance between reactive oxygen forms and the ability of a cell to neutralize their damaging effects through redox reactions. As a result, free radicals and superoxides damage cellular components and are especially harmful to proteins, lipids, and DNA. In healthy individuals, the retina has the greatest oxygen consumption per weight of any organ in the body, making it naturally vulnerable to OS [3,31,32]. In our study, we identified a number of proteins involved in OS induction and management in tear samples isolated from neovascular AMD patients. One of the most striking findings was the downregulation of glutathione S-transferase P. Glutathione (GSH) is one of the most important, ubiquitous antioxidant agents, whose role in the retinal anti-OS defense is well-established. It works by scavenging the reactive oxygen species and is a cofactor for GSH S-transferase P [9,10]. Its lowered concentration in the tear film of AMD patients may suggest the impaired cell-detoxification mechanisms.

Aldo-keto reductase family 1 member A1 (AKR1A1) is yet another protein involved in the cellular protection against OS, which was downregulated in our study, but its connection with the retina remains unclear [11,12]. 

Additionally, the identified protein retinal dehydrogenase 1 (RALDH1) is involved in redox reactions. Its key function is to oxidize retinaldehyde into retinoic acid, which participates in cell growth and differentiation and plays a critical role in the visual cycle [13].

Overall, the findings suggest that selected OS biomarkers can be found in tears from neovascular AMD patients.

### 4.2. Protein Clearance

Increased OS can damage proteins, and damaged proteins must be removed to prevent intracellular protein aggregation. Protein clearance impairment plays a crucial role in AMD development. Under normal conditions, retinal cells maintain proteostasis through two major mechanisms: proteasome-mediated degradation and lysosome-mediated autophagy. In AMD, impairment of phagocytosis leads to failure of the degradation of photoreceptor outer segments (POSs) in lysosomes, while impairment of autophagy leads to the accumulation of toxic protein aggregates and organelles, such as mitochondria [14]. Since RPE cells are quiescent cells, the consequences of deficient proteostasis are potentially devastating [15]. 

Annexin A1 and A4 are a part of the calcium-dependent phospholipid-binding family. Annexins A, beside regulating the inflammatory process described above, are vitally important to the autophagy process, and take an important part in the formation of the cytoskeleton, cell membrane, and in the cell signaling [16]. Annexin A1 is involved in the autophagosome-lysosome fusion, and its upregulation seems to inhibit autophagy process via PI3K/AKT activation followed by Beclin-1 and ATG5-dependent autophagy inhibition [17,18]. This may lead to the pathological aggregation of debris material within the RPE-BM complex, called drusen, and further stimuli for the formation of the choroidal neovascularization (CNV).

Given all of the above findings, the upregulation of the Annexin A1 and A4 in the tears of patients with neovascular AMD may indicate that proteostasis is disturbed in AMD.

### 4.3. Chronic Inflammation and Neovascularization

Increased inflammation is well established to occur during AMD pathogenesis in response to chronic OS and disturbed proteostasis [14]. Short lasting inflammation is a beneficial host defense in cells, while prolonged inflammation of low intensity (parainflammation) can lead to CNV and cell death in the context of AMD [19,20].

Annexin A1 (ANXA1) was found in our study on three different electrophoretic spots, probably to its further post treatment modification, which suggests its strong presence in the AMD tear film. Its concentration in the samples obtained from AMD patients was almost 5 times higher than in the control group. Previous studies investigating the ANXA1 impact on inflammation proved its significant anti-inflammatory potential [21,22,23]. It was also already found in the aqueous humor of the wet AMD patients (both Annexin A1 and A4) [24], in the drusen from the donors retina [25]. The impairment of the Annexins family function is related to numerous diseases, also neurodegenerative disorders and glaucoma, although in none it seems to be a primary cause [26,27,28].

Alpha-Enolase, which was found to be upregulated in our AMD patients’ group, is another protein that can act as an autoantigen in the autoimmune process, which was already connected with AMD. Elevated levels of the antibodies against α-enolase were found in AMD patients’ serum [29,30]. It is also strongly connected with the development of cancer-associated retinopathy (CAR), Alzheimer’s disease (AD), cancer, and other diseases [31,32,33].

Another protein directly involved in inflammation and neovascularization process is allograft inflammatory factor 1 (AIF-1). In mouse models of neovascular AMD, it was highly expressed in an induced laser scarring spot, leading to NF-κB activation, and further CNV development [34]. It is also an established biomarker in local immune and inflammatory response of the retinal cells [35,36,37]. The questionable aspect is the downregulation of AIF-1 observed in our study, one would expect it to be upregulated.

Another hallmark of AMD is choroidal neovascularization (CNV), in which vessels sprout from the choroid and pass through the BM and the RPE, causing subretinal leakage, macular edema, and hemorrhages. In the end stage, a disciform scar is formed, with mesenchymal transition of RPE cells and general retinal disorganization. One of the key modulators of this process is VEGF, and the treatment of choice is anti-VEGF delivered via intravitreal injections. Although anti-VEGF administration has been a major breakthrough in AMD treatment, it has significant limitations: continued visits are necessary, macular scarring can occur, and patients can be refractory to treatment [38]. One of the proteins crucial for the BRB development, ATP-dependent translocase ABCB1, was found to be upregulated in our study. ABCB1 is responsible for the cellular transport, being an efflux pump, and is commonly associated with various types of cancers, due to its role in the multidrug resistance (MDR) [39,40,41].

Scarring is the eventual effect of the CNV presence, whether due to the treatment or the natural course of the disease. Elongation factor 2 was found to be downregulated in our AMD group. This protein was also identified in the Müller glia in course of the proliferative vitreoretinopathy, which is essentially a scarring process [42]. Elongation factors were also found to be downregulated in older retinas, which can partially explain its downregulation in our study [43].

Last of the identified proteins, short stature homeobox 2 has not been yet described in the context of AMD, or retinal dysfunction, and it should probably be concerned as an accidental finding. It was recently found that it may serve as a biomarker for bronchial squamous cell carcinoma (SCC) [44].

All these findings suggest a strong inflammatory component that can be observed in the tear film of AMD patients, which stays in line with our current knowledge of the disease.

### 4.4. Anti-VEGF Treatment

All of the patients included in our study were treated with intraocular anti-VEGF injections over the course of a national drug program. This warranted quality patient selection and confirmation of medical history. The patients were first qualified by a local ophthalmologist, and then the diagnosis was confirmed online by nationally board-certified retinal AMD specialists. In all cases, the samples were collected at the follow-up day before the anti-VEGF injection. Therefore, each patient was examined 28 to 31 days after previous anti-VEGF treatment. Since we did not include treatment-naïve patients with AMD, anti-VEGF treatment might have affected the protein expression results. This may explain why we did not observe differential expression of certain proteins, such as VEGF or PDGF, even though differences in such proteins have been found in other studies [45,46]. On the other hand, this might have enabled the roles and differential expression of other proteins involved in neovascularization. The VEGF pathway, although extremely important, is certainly not the sole promotor of the growth of the new vessels. Preferably, a larger clinical study should be conducted on samples collected from patients at different stages of AMD progression.

### 4.5. Limitations of the Study

One major limitation of our study is the correlation between the tear film composition and macular lesions. Although the tear film is not directly connected with the retina, it can be altered by the partial blood–retinal barrier breakdown (BRB) in the course of AMD. BRB was mainly described in diabetic retinopathy, and functions as a key factor in this disease, but the BRB can also be found in the neovascular AMD, where macular edema, subretinal fluid, and vitreous hemorrhages are present [47,48,49,50]. Thus, we believe that in an active phase of neovascular AMD, it is possible that the leakage from the pathological vessels can be also detected in the tear film.

Another limitation of this study is that being a pilot study, we were not able to analyze enough samples to reach adequate power of the tests used in statistical calculations. According to an amount of wet AMD cases in our region, we would need over 300 samples for each group. This will be done in the following experiments. 

## 5. Conclusions

Tear film is a well-established material for obtaining biomarkers of various diseases. We believe that the findings of this study enhance the current understanding of AMD as a multifactorial disease with underlying persistent OS, cell clearance mechanism impairment, inflammation, and CNV. Although the identified proteins probably should not be considered verified biomarkers, the differences in their expression between groups suggest that they are connected to ongoing pathological processes in the macula and tear film. Further studies are needed to confirm this possibility, preferably studies comparing the levels of specific proteins in different body fluids, such as the plasma, aqueous humor, and tear film.

## Figures and Tables

**Table 1:** Significantly (*p* ≤ 0.05) differentially expressed proteins in neovascular age-related macular degeneration (AMD) patients as identified by MALDI-TOF MS. Listed molecular weights and pI values correspond to the MASCOT search results; carbamidomethylation of cysteine was a global modification. Rt (Ratio) quotient of the group means of relative spot volumes; volume of a given spot in control group is the denominator of the ratio parameter.

ID	Protein	Accession Number (UniProtKB)	Species	Score	Match	MW (Da)	pI	Seq. Cov (%)	Rt	*p*-Value
1	ATP-dependent translocase ABCB1	P08183	*H. sapiens*	87	11	141,788	9.06	9	2.193	0.025
5	Annexin A1	P04083	*H. sapiens*	96	12	38,918	6.57	41	0.664	0.026
6	Annexin A1	P04083	*H. sapiens*	59	9	38,918	6.57	30	0.575	0.017
8	Aldo-keto reductase family 1 member A1	P14550	*H. sapiens*	146	15	36,892	6.32	48	0.638	0.029
10	Retinal dehydrogenase 1	P00352	*H. sapiens*	75	9	55,454	6.30	24	2.027	0.011
12	Uncharacterized protein C11orf98	E9PRG8	*H. sapiens*	76	5	14,225	11.53	38	0.560	0.008
15	Glutathione S-transferase P	P09211	*H. sapiens*	89	8	23,569	5.43	50	0.529	0.007
23	Retinal dehydrogenase 1	P00352	*H. sapiens*	121	14	55,454	6.30	41	1.991	0.015
24	Alpha-enolase	P06733	*H. sapiens*	67	11	47,481	7.01	29	1.476	0.022
11	Annexin A4	P09525	*H. sapiens*	94	14	36,088	5.84	14	0.393	0.003
21	Annexin A1	P04083	*H. sapiens*	76	11	38,918	6.57	35	0.213	0.008
31	Allograft inflammatory factor 1	P55008	*H. sapiens*	77	5	16,693	5.97	34	0.560	0.026
33	Cytospin-A or Elongation factor 2	Q69YQ0 P13639	*H. sapiens*	113 88	16 12	124,925 96,246	5.52 6.41	16 12	0.560	0.037
32	Short stature homeobox protein 2	O60902	*H. sapiens*	65	5	35,160	8.99	12	0.529	0.041

Abbreviations: MW—molecular weight; pI—isoelectric point; Seq. Cov—sequence coverage; Rt—ratio.

**Table 2:** AMD group up- and downregulated proteins.

Identified Protein	Upregulation or Downregulation	Fold Relative to Healthy Controls	Standard Deviation (SD)
Retinal dehydrogenase 1	Up	2.072 1.991	0.011 0.015
ATP-dependent translocase ABCB1	Up	2.193	0.025
Alpha-enolase	Up	1.476	0.022
Annexin A1	Down	0.664 0.575 0.213	0.026 0.017 0.008
Annexin A4	Down	0.393	0.003
Aldo-keto reductase family 1 member A1	Down	0.638	0.029
Uncharacterized protein C11orf98	Down	0.560	0.008
Glutathione S-transferase P	Down	0.529	0.007
Allograft inflammatory factor 1	Down	0.560	0.026
Cytospin-A or Elongation factor 2	Down	0.560	0.037
Short stature homeobox protein 2	Down	0.529	0.041

## Data Availability

Data available after contacting corresponding author: mateuszwiniarczyk@umlub.pl.

## Supplementary Materials

Supplementary materials can be found at https://www.mdpi.com/article/10.3390/jcm10143060/s1. Supplementary Table S1: Characteristics of wet AMD patients. Supplementary, Table S2: Characteristics of control group patients.
