# Supplementary material for: Dysregulated Tear Film Proteins in Macular Edema Due to the Neovascular Age-Related Macular Degeneration Are Involved in the Regulation of Protein Clearance, Inflammation, and Neovascularization
Source: J Clin Med. 2021 Jul 10;10(14):3060. doi: 10.3390/jcm10143060 (PMC8307956; doi:10.3390/jcm10143060)
Supplement: Supplementary file 1 [file jcm-10-03060-s001.zip › jcm-1257523-supplementary.pdf]

Table S1. Characteristics of wet AMD patients.

Sex/age	BCVA OD/OS (Snellen)	Schirmer test result OD/OS	SRF presence	Number of previous anti-VEGF injections	Ocular interventions	Systemic diseases	Smoking
**F/76**	0.2/0.4	14/14	+	3	CS (2)	HT	Y
**F/81**	0.05/0.7	19/20	+	6	CS (2)	CVD,HT	Y
**F/75**	0.6/0.3	20/20	+	2	0	-	Y
**F/88**	0.5/0.5	16/17	+	7	CS (1)	HT, CVD	Y
**F/69**	0.2/0.1	18/14	+	3	CS (2)	-	N
**F/71**	0.4/0.4	19/17	+/-	3	0	-	Y
**F/80**	0.2/0.5	17/17	+	6	CS (1)	HT	N
**F/78**	0.7/0.3	20/20	+	10	0	HT	N
**M/80**	0.6/0.2	16/16	+	5	0	HT,CVD	N
**M/82**	0.8/0.05	15/16	+	8	CS (2)	HT,CVD	Y
**M/77**	0.4/0.4	20/20	+	6	CS (2)	HT	Y
**M/70**	0.1/0.05	19/15	+	9	0	HT	Y
**M/79**	0.5/0.8	13/12	+	4	CS (1)	HT	Y
**M/68**	1.0/0.6	20/20	+	2	CS (1)	HT,CVD	Y
**M/72**	0.3/0.5	20/20	+	6	CS (2)	-	Y

Abbreviations: F- female; M- male; BCVA – best corrected visual acuity; OD- oculus dexter (right eye); OS- oculus sinister (left eye); SRF- subretinal fluid; CS – cataract surgery; HT- hypertension; CVD-cardiovascular disease; Y – yes; N - no.

Table S2. Characteristics of control group patients.

Sex/age	BCVA OD/OS (Snellen)	Schirmer test result OD/OS	Ocular interventions	Systemic diseases	Smoking
**F/75**	1.0/0.4	13/17	CS (1)	-	N
**F/77**	0.6/0.5	11/12	0	HT	Y
**F/69**	0.8/0.9	16/16	CS (2)	-	Y
**F/80**	0.4/0.7	16/16	CS (1)	HT, CVD	Y
**F/78**	0.6/0.2	20/20	0	HT	N
**F/76**	0.7/0.5	19/20	0	HT	Y
**F/79**	0.5/0.5	16/18	0	-	N
**M/75**	0.3/0.7	20/18	0	HT	Y
**M/75**	0.4/1.0	18/16	CS (1)	HT,CVD	Y
**M/76**	1.0/0.7	17/17	CS (1)	-	Y
**M/75**	1.0/0.8	18/19	0	HT	N
**M/86**	0.7/0.7	12/12	0	HT, CVD	Y
**M/75**	1.0/1.0	16/17	CS (1)	HT	Y
**M/74**	0.8/0.9	16/18	CS (2)	HT, CVD	Y
**M/71**	0.2/1.0	20/18	CS (1)	HT, CVD	Y

Abbreviations: F- female; M- male; BCVA – best corrected visual acuity; OD- oculus dexter (right eye); OS- oculus sinister (left eye); SRF- subretinal fluid; CS – cataract surgery; HT- hypertension; CVD-cardiovascular disease; Y – yes; N - no.
